# Structural Basis for Specific Binding of Human MPP8 Chromodomain to Histone H3 Methylated at Lysine 9

**DOI:** 10.1371/journal.pone.0025104

**Published:** 2011-10-12

**Authors:** Jing Li, Zhihong Li, Jianbin Ruan, Chao Xu, Yufeng Tong, Patricia W. Pan, Wolfram Tempel, Lissete Crombet, Jinrong Min, Jianye Zang

**Affiliations:** 1 Key Laboratory of Structural Biology, Chinese Academy of Sciences, and School of Life Sciences, University of Science and Technology of China, Hefei, Anhui, People's Republic of China; 2 Structural Genomics Consortium, University of Toronto, Toronto, Ontario, Canada; 3 Department of Physiology, University of Toronto, Toronto, Ontario, Canada; University of Cambridge, United Kingdom

## Abstract

**Background:**

M-phase phosphoprotein 8 (MPP8) was initially identified to be a component of the RanBPM-containing large protein complex, and has recently been shown to bind to methylated H3K9 both *in vivo* and *in vitro*. MPP8 binding to methylated H3K9 is suggested to recruit the H3K9 methyltransferases GLP and ESET, and DNA methyltransferase 3A to the promoter of the E-cadherin gene, mediating the E-cadherin gene silencing and promote tumor cell motility and invasion. MPP8 contains a chromodomain in its N-terminus, which is used to bind the methylated H3K9.

**Methodology/Principal Findings:**

Here, we reported the crystal structures of human MPP8 chromodomain alone and in complex with the trimethylated histone H3K9 peptide (residue 1–15). The complex structure unveils that the human MPP8 chromodomain binds methylated H3K9 through a conserved recognition mechanism, which was also observed in *Drosophila* HP1, a chromodomain containing protein that binds to methylated H3K9 as well. The structure also reveals that the human MPP8 chromodomain forms homodimer, which is mediated via an unexpected domain swapping interaction through two β strands from the two protomer subunits.

**Conclusions/Significance:**

Our findings reveal the molecular mechanism of selective binding of human MPP8 chromodomain to methylated histone H3K9. The observation of human MPP8 chromodomain in both solution and crystal lattice may provide clues to study MPP8-mediated gene regulation furthermore.

## Introduction

Histones are subject to a wide variety of posttranslational modifications including acetylation, methylation, phosphorylation, ubiquitination, sumoylation and so on [1]. These post-translational modifications (PTM) constitute ‘histone code’, which will be read in part by histone PTM-binding ‘effector’ modules and their associated complexes [2], [3], [4]. Lysine methylation of histone tail has been known for more than 30 years [3], [5]. Currently, numerous studies have revealed that a number of domains could bind methylated histone tails, including WD40 repeats [6], PHD fingers, Ankyrin repeats, MBT domain [7], [8], Tudor domain, Chromodomain, PWWP domain and chromo barrel domains [7], [9], [10], [11], [12], [13], [14], [15], [16], [17], [18], [19], [20], [21], [22], [23], [24]. The common feature of the recognition is that the methylated lysine residue is coordinated via a conserved aromatic cage around the moiety. Chromodomain was first identified as methyllysine binding motif in *Drosophila melanogaster* heterochromatin protein-1 (HP1) and Polycomb as regulators of chromatin structure that are involved in epigenetic repression [25], [26]. The structures of the HP1 chromodomain in complex with a methyl-Lys 9 histone H3 peptide and the Polycomb chromodomain in complex with a methyl-Lys 27 histone H3 peptide reveal the molecular mechanism of chromodomain binding to methylated histone H3 [23], [24], [27]. Many other chromodomain-containing proteins, such as CHD1, Eaf3, MSL3, MPP8 and so on, were also reported to recognize methylated histone tails [28], [29], [30], [31]. Most chromodomain-containing proteins participate in the formation of large multiprotein complexes to facilitate their recruitment to target loci, resulting in chromatin remodeling and transcription repression [32].

The M-phase phosphoprotein 8 (MPP8), which was firstly identified to coimmunoprecipitate with the RanBPM-comprised large protein complex, was shown to associate with methylated H3K9 both *in vivo* and *in vitro* [33], [34], [35]. The binding of MPP8 to methylated H3K9 recruited the H3K9 methyltransferases GLP and ESET, as well as DNA methyltransferase 3A (DNMT3A) to the promoter of the E-cadherin gene, a key regulator of tumor cell growth and epithelial-to-mesenchymal transition (EMT) [36], [37]. The recruitment of those enzymes and enzyme complexes, which regulated the H3K9 and DNA methylation at the promoter of E-cadherin gene, respectively, repressed the tumor suppressor gene expression and, in turn, played an important role in epithelial-to-mesenchymal transition and metastasis [34].

Here, we reported the crystal structures of human MPP8 (hMPP8) chromodomain both in free form and in complex with the trimethylated histone H3 lysine 9 (H3K9me3) peptide (residue 1–15). Consistent with the high sequence homology of MPP8 with Polycomb and HP1 chromodomains, the complex structure of hMPP8-H3K9me3 uncovers the detailed molecular mechanism of recruitment of MPP8 chromodomain by HK9me3 as well as its unexpected homodimerization. In this way, our study sheds lights on the roles of MPP8 in regulating gene expression.

## Results

### Overall structure of hMPP8 chromodomain

To unveil the molecular architecture of the chromodomain of hMPP8, hMPP8 chromodomain (55–116 residues) was recombinantly expressed and crystallized. The crystals of the free-hMPP8 and hMPP8-H3K9me3 complex both diffracted to 2.05 Å resolution and the structures were solved using molecular replacement. The quality of the X-ray diffraction data and the structure refinement parameters are shown in Table 1.

**Table 1:** Data collection, phasing and refinement statistics for MPP8 and MPP8-H3K9me3 complex.

PDB code	3LWE	3R93
**Data collection**		
Crystal	MPP8	MPP8-H3K9me3
Space group	*P*6_5_	*P*2_1_2_1_2
Cell dimensions		
a, b, c (Å)α,β,γ(°)	50.66, 50.66, 123.5490, 90, 120	71.15, 74.00, 72.6190, 90, 90
Wavelength (Å)	0.9794	0.9792
Resolution range (Å)	50.00-2.05 (2.09-2.05)^a^	50.00-2.05 (2.12-2.05)
Unique reflections	11,280 (551)	24,361 (2,340)
Mutiplicity	11.3 (11.2)	6.8 (5.0)
Data completeness (%)	99.7 (99.6)	99.7 (97.6)
R_merge_ (%)^b^	7.6 (85.3)	7.2 (73.5)
*I*/σ (*I*)	38.5 (3.9)	34.8 (2.1)
**Refinement statistics**		
Resolution (Å)	43.88-2.05	30.00-2.06
No. of reflections R_work_/R_free_	11,249/537	23,004/1,237
No. atoms	1,062	2,269
Protein	995	1,838
Peptide	0	340
Water	67	91
R_work_ (%)	21.7	22.0
R_free_ (%)	28.3	27.3
R.m.s.d. bond length (Å)	0.018	0.014
R.m.s.d. bond angle (°)	1.6	1.4
Mean B-value (Å^2^)	36.8	51.2
Protein	36.6	50.5
Peptide	n/a	56.6
Water	40.4	46.8
Ramachandran plot (%) (favored/additional/disallowed)^C^	92.6/7.4/0.0	93.0/7.0/0.0

a Values in parentheses are for highest-resolution shell.

b R_merge_ = Σ_hkl_Σ_i_ I*I*_i_(hkl)−<I(hkl)>I/Σ_hkl_Σ_i_ *I*_i_(hkl), where *I*_i_(hkl) is the ith observation of reflection hkl and <I(hkl)> is the weighted average intensity of all observations i of reflection hkl.

c Statistics for the Ramachandran plot from an analysis using Procheck.

**Figure 1:**
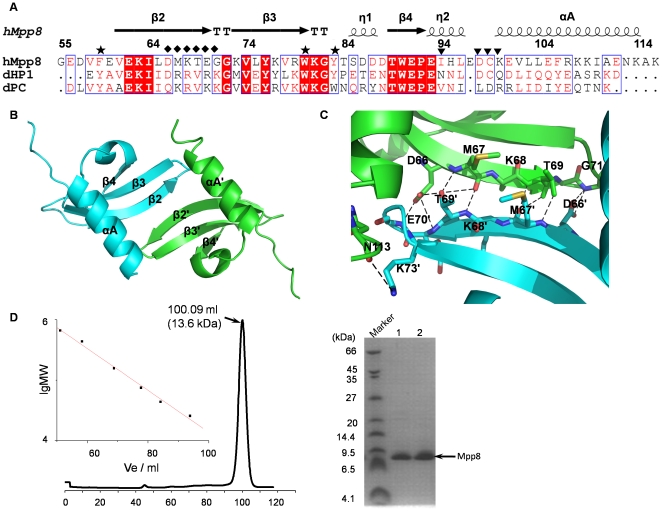
Overall structure of hMPP8 chromodomain. (A) Sequence alignment of chromodomains of human MPP8, *Drosophila* HP1 and *Drosophila* Polycomb. Secondary structural elements (arrows for β strands and rectangles for α helices) are indicated, η represent 3_10_ helix. Methylated H3K9 binding residues are marked by stars. Residues involved in dimerization of human Mpp8 and *Drosophila* Polycomb are marked by diamonds and triangles, respectively. The alignment was created with Espript (http://espript.ibcp.fr/Espript/Espript). (B) Cartoon representation of the crystal structure of hMPP8 chromodomain. The two subunits of the homodimer are colored in cyan and green, respectively, and the secondary structure regions in both proteins are marked. (C) Interactions between the two subunits of the hMPP8 chromodomain homodimer. The dimer interface is formed by strand β2 through an antiparallel arrangement. (D) Gel filtration of hMPP8 chromodomain (left panel). The column buffer was 20 mM Tris (pH 8.0), 400 mM NaCl. The molecular weight of hMPP8 chromodomain monomer was measured using tricine-SDS-PAGE (right panel).

In the free form, the hMPP8 chromodomain consists of a twisted anti-parallel β-sheet formed by three β-strands (named β2–β4), and α helix (named αA) located at the C-terminal end packing against one edge of the β-sheet next to β2 (Fig. 1B). In the asymmetric unit of the crystal, two hMPP8 chromodomain monomers form a dimer through the interaction between the β2 strand from each monomer. The β2 strand from one subunit runs anti-parallel to the β2' strand from the neighboring one, pulling the three-stranded anti-parallel β-sheets of two hMPP8 chromodomain proteins adjacent to constitute a six-stranded anti-parallel β-sheet (Fig. 1B). Specifically, Asp66, Met67, Thr69, Gly71 and Gly72 of β2 strand form hydrogen bond with Gly72', Gly71', Thr69', Met67' and Asp66' of β2' strand from the opposite subunit, respectively. In addition, Asp66, Met67, Lys68, Glu70, Lys109, Ile110 and Asn113 contact Thr69', Glu70', Met67', Asn113' and Ile110' via van der waals interactions (Fig. 1C). The dimer interface has a buried surface area of about 1025 Å^2^, which is strong enough to form a stable dimer. As reported, the dHP1 chromodomain existed as monomer while dPlycomb chromodomain formed dimer both in solution and in crystal lattice [19], [20], [23], [30]. Sequence alignment result indicates that hMPP8 chromodomain is more similar to HP1 choromodomain and lacks the residues in dPlycomb chromodomain for dimerization (Fig. 1A). It was quite unexpectedly to find that hMPP8 chromodomain forms homodimer in our crystal structures.

To determine the oligomerization state of hMPP8 chromodomain in solution, size exclusion experiment was performed. As shown in Fig. 1D, hMPP8 chromodomain eluted as a single peak with apparent molecular weight of 13.6 kD. The molecular weight of hMPP8 chromodomain monomer is about 8.0 kD. The size of hMPP8 chromodomain in solution is corresponding to dimer, consistent with the observation in crystal structure. Therefore, the chromodomain exists as a homodimer in solution (Fig. 1D) and the homodimer structure in crystal is not due to crystal packing.

### Structural basis for the specific binding of the hMPP8 chromodomain to histone H3 methylated at lysine 9

**Figure 2:**
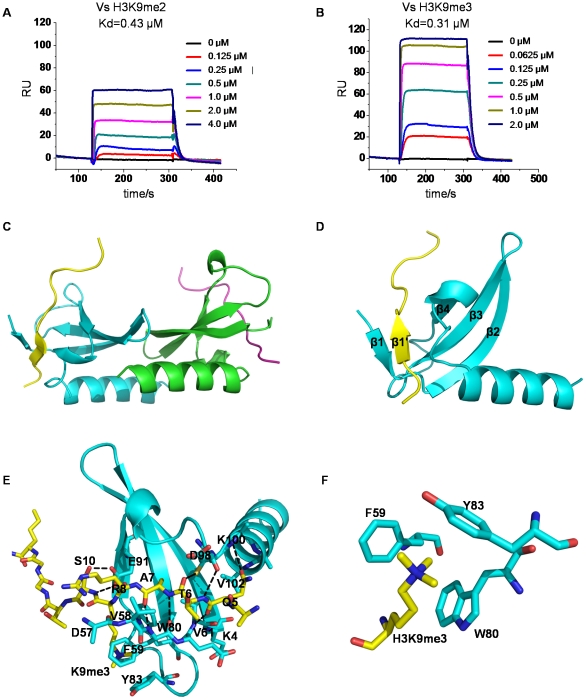
hMPP8 chromodomain specifically recognizes di- and tri- methylated H3K9 peptides. (A) and (B) Binding affinity of hMPP8 chromodomain to di- and tri-methylated H3K9 peptides was measured by SPR method (C) Overall structures of hMPP8 chromodomain in complex with histone H3K9me3 peptide. Cyan and green: hMPP8 chromodomain, yellow and magenta: methylated histone H3K9 peptide. (D) Monomer structure of hMPP8 chromodomain in complex with histone H3K9me3 peptides. Cyan: hMPP8 chromodomain, yellow: histone H3K9me3 peptide. (E) Interactions between hMPP8 chromodomain and H3K9me3 peptide. The chromodomain is shown in cartoon representation and colored in cyan. The H3K9me3 peptide is shown in a stick mode. (F) The aromatic cage accommodating trimethylated lysine 9.

Since hMPP8 chromodomain was reported to bind methylated H3K9 [34], [35], [38], we used synthetic di- and tri-methylated H3K9 (residues 1 to 15) peptides to measure their binding affinities to the hMPP8 chromodomain by surface plasmon resonance (SPR) method. The hMPP8 chromodomain showed strong binding to both di- and tri- methylated H3K9 with the dissociation constants of 0.43 µM and 0.31 µM, respectively (Fig. 2A, 2B). In histone H3, the amino acid sequence around lysine 27 site (KAARK^27^S) is similar to that of the lysine 9 site (QTARK^9^S). A synthetic H3K27me3 (residues 19 to 33) peptide was also used to determine the binding affinity to hMPP8 chromodomain. However, the hMPP8 chromodomain does not exhibit detectable binding to the both H3K27me3 peptide (Fig. S1F). In addition, the binding of hMPP8 chromodomain to H3K4me3 peptide was unable to be detected (Fig. S1F), which was consistent with previous reports [31], [35]. To explore how the hMPP8 chromodomain selectively binds the methyl-K9-containing histone H3 tail, we determined the crystal structure of the hMPP8 chromodomain in complex with the H3K9me3 peptide. The overall structure of the hMPP8 chromodomain in complex with the H3K9me3 peptide is shown in Fig. 2C. Two histone H3K9me3 peptides bind to the opposite faces of the hMPP8 chromodomain homodimer, respectively (Fig. 2C). Structural comparison of the hMPP8 chromodomain-H3K9me3 peptide complex and the free hMPP8 chromodomain identified a newly formed β strand (named β1) by the N-terminal residues, which exited as a loop in the free hMPP8 chromodomain structure (Fig. 2D). This β strand is induced by the contact with the H3 tail peptide, which was observed in the structures of *Drosophila* HP1 and Polycomb chromodomain in complex with methyllysine histone peptides before [23], [24], [27]. From the complex structure we can see that the H3K9me3 peptide binds to hMPP8 chromodomain in a cleft between the N-terminal newly formed β1 strand and the loop connecting β4 and αA. Similar to the structure of the *Drosophila* HP1 and Polycomb chromodomain in complex with methyllysine histone peptides, the interactions between hMPP8 chromodomain and H3K9me3 largely involve the main chains of both the protein and the peptide, including the residues Gln5, Thr6, Ala7, and Arg8 of the H3 tail and the residues Val58, Phe59, Glu60, and Val61 located at the β1 strand in hMPP8 chromodomain. In addition, the residues of Gln5 and Arg8 form van der waals contacts with the residues of 98–100 located in the loop connecting β4 and αA, whereas Gln5 and Ser10 form hydrogen-bonds with residues of Glu101, Val102 and Glu91, respectively (Fig. 2E). As demonstrated in most complex structures of methyllysine peptides and their recognition modules, the trimethylated K9 lies in a hydrophobic pocket formed by three aromatic residues, Phe59, Trp80, and Tyr83 (Fig. 2F). And the trimethyl-K9 is anchored by cation-π and van der Waals interactions within this aromatic cage.

It is noteworthy that the sequence motif of H3K27 is similar to H3K9 tail (KAARK^27^S versus QTARK^9^S). However, methylated histone H3K27 cannot interact with hMPP8 chromodomain. To explain why hMPP8 specifically recognizes methylated H3K9, we built a mutant model that hMPP8 binds to methylated H3K27. We mutated residues Gln5 and Thr6 of the QTARK^9^S motif to KA to generate a motif of KAARK^9^S, which shared the same amino sequence around the K27 site in histone H3. In this mutant model, we found that the side chain of Lys5 prevented the insertion of histone peptide into the binding groove of hMpp8 chromodomain (Fig. S1A). To further validate the hypothesis, hMpp8 chromodomain mutants were designed to rescue the binding ability to H3K27me3. Based on our structures, residues Lys100, Glu101 and Val102 were mutated to proline, respectively, to generate enough space where the side chain of Lys5 can insert. As expected, when Lys100 or Glu101 were mutated to proline respectively, the mutant proteins were found to be able to bind H3K27me3 peptide weakly (Fig. S1B).

Furthermore, a structure model of peptide KAARK(me3)S (referred to as H3K27me3 peptide), which shared the same amino sequence around the K27 site in histone H3 was generated and docked into hMPP8 chromodomain structure using the program HADDOCK [39] (Fig. S1C). In this model, the H3K27me3 peptide can still form a β sheet and interact with hMPP8 via hydrogen-bond and van der Waals interactions. However, the side chain of trimethyllysin was pushed about 24° away from the original binding site in the hydrophobic pocket, which is essential for the interaction between methylated peptide and its association domain (Fig. S1D). Thus we believe that altering the motif of QT to KA abolishes the binding ability of H3K27me3 peptide to hMPP8 chromodomain.

### Structural comparison of hMPP8 with HP1 and Polycomb chromodomains

**Figure 3:**
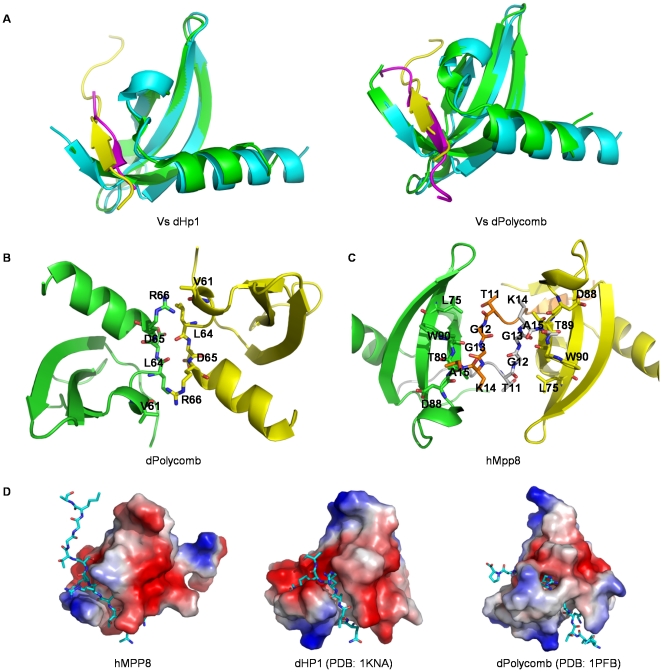
Structural comparison of hMPP8, HP1 and Polycomb chromodomains. (A) Superimposition of hMPP8 (cyan) with *Drosophila* HP1 (green), and hMPP8 (cyan) and *Drosophila* polycomb (green) chromodomain in complex with methylated histone H3K9 peptide (yellow and magenta, respectively). (B) The *Drosophila* polycomb chromodomain dimer. Two *Drosophila* polycomb chromodomain monomers are shown in a cartoon representation, colored yellow and green, respectively. Key residues involved in dimerization are shown in a stick mode. (C) Histone-histone and histone-chromodomain interactions of hMPP8 chromodomain in complex with H3K9me3 peptide in crystal lattice. Chromodomains are shown in a cartoon representation, colored yellow and green, respectively. Key residues involved in interactions are shown in a stick mode. (D) Peptide binding grooves of hMPP8, dHP1 and dPolycomb chromodomains. Chromodomain is shown in surface representation, and histone peptide is shown in a stick mode.

Consistent with the high sequence homology of the hMPP8 chromodomain to the *Drosophila* HP1 and Polycomb chromodomains (Fig. 1A), the overall structure of hMPP8, Polycomb and HP1 chromodomains are very similar [23], [24], [27]. Unsurprisingly, the binding mode of hMPP8 to the methylated-H3K9 peptide is also similar to that observed in the structures of HP1 and Polycomb chromodomain in complex with the methylated histone peptides. The structure of hMPP8 chromodomain is well conserved with an RMSD of 0.8 Å and 1.1 Å for all aligned C_α_ atoms with those of the HP1 and Polycomb chromodomain, respectively. In addition, the histone peptide conformation in the hMPP8 chromodomain complex structure is also very similar to its counterparts in the complex structures of the HP1 and Polycomb chromodomains, with an RMSD of 0.4 Å and 0.5 Å, respectively (Fig. 3A). Though the architectural features of the hMPP8, HP1 and Polycomb chromodomains are highly similar, there are still many noticeable differences among them.

The most striking difference is that the hMPP8 chromodomain was found to form homodimer in both solution and crystal lattice, which was not observed in *Drosophila* HP1. Although self-association of the chromodomain of *Drosophila* Polycomb has been pointed out explicitly before [27], the interaction mode of the two subunits in hMPP8 chromodomain homodimer is different from that of Polycomb. In Polycomb chromodomain homodimer, the key residues that involved in dimerization are located in the loop at C-terminus connecting the last β strand and the last α helix (Fig. 1A, 3B). However, dimerizaiton of hMPP8 chromodomain is formed via extensive intermolecular interactions between the two β2 stands from two individual subunits, including van der waals contacts and hydrogen-bonds. (Fig. 1C).

A total of 12 out of 15 residues in H3K9me3 were observed to be ordered in hMPP8 chromodomain complex corresponding to the sequence stretch from Lys 4 to Ala 15, whereas there were only 6 and 9 residues observed in the structures of *Drosophila* HP1 and Polycomb, respectively [23], [24], [27]. hMPP8 chromodomain possesses a more extended peptide binding groove than that of HP1, comparing the 1250 Å^2^ of the interaction area of hMPP8 to 1063 Å^2^ of HP1. Nevertheless, it is not convincing to deduce that the longer H3 tail observed in the structure of hMPP8 chromodomain complex is just because of the extended protein-peptide interaction, since Polycomb chromodomain has the most extended peptide binding groove among the three, 1482 Å^2^ (Fig. 3D). In the crystal lattice, another pattern of the homodimer of hMPP8 chromodomain was found. The two chromodomain juxtaposed the two H3- binding clefts in an antiparallel fashion and resulted in not only histone-histone interactions involving Ser10, Gly12 and Cly13 of H3, but also the interactions between histone peptide and the neighboring chromodomain involving residues 11–15 of H3 with residues leu75 and 88–90 of the adjacent chromodomain. Those additional interactions can further stabilize H3 peptide, especially residues 11–15 (Fig. 3C).

To verify whether the H3K9me3 peptide could bring the two chromodomain homodimers together in solution, size-exclusion chromatography were performed to determine the oligomerization state of hMPP8 chromodomain either in the presence or in the absence of H3K9me3 peptide. The elution volumes of hMPP8 chromodomain in free form and in complex with H3K9me3 peptide were approximately 12.62 ml and 12.60 ml, respectively, which were both corresponding to the homodimer of hMPP8 chromodomain (Fig. S1E). The results indicated that the pattern that two chromodomain juxtaposed the two H3-binding clefts in neighboring hMPP8 chromodomain dimers is only a crystal-packing artifact.

## Discussion

Recently, more and more evidences have suggested that many histone-mark “readers” and “writers” can also bind non-histone sequences [40], [41], [42]. Chromodomain is conserved among both plants and animals, which functions individually or in tandem to recognize specific methylated histone tails [43], [44]. CBX3 chromodomain have been reported to bind H3K9me3 peptide [45]. However, a recent structure of CBX3 chromodomain in complex with G9a peptide (PDB: 3DM1) demonstrates that CBX3 is also a reader of methylated G9a. Here, we resolve the structure of hMPP8 chromodomain in complex with H3K9me3 peptide and shed lights on the molecular mechanism of selective binding of hMPP8 to methylated histone H3K9. Based on our structure, we tried to mutate some residues of H3K9me3 peptide and generate structure models by docking the mutant peptides into the hMpp8 chromodomain using the program HADDOCK [39], we finally hypothesized a consensus sequence of (Q/N)(T/V/L/I/S)A(R/K/H)Kme(S/T) (“/” separates tolerated amino acids at each site). Such consensus sequence may be helpful to predict the candidate Mpp8-interacting proteins which could potentially be methylated.

In addition, our crystal structures reveal that hMpp8 forms homodimer via β-sheet interactions between the neighboring subunits, which are never observed in the structure of either HP1 or Polycomb chromodomain before. The distanance between the two aromatic cages binding methylated H3K9 in hMpp8 chromodomain homodimer is measured to be 40 Å, so it would be reasonable to speculate that hMPP8 chromodomain dimer may bind two methylated H3K9 from the same nucleosome or spatially adjacent nucleosomes. Here we build the models that the simultaneous binding of two histone tails to hMpp8 homodimer either from the same nucleosome or from two separated nucleosomes (Fig. S2A and S2B). We believe that the interactions of hMPP8 homodimer with two histone H3 tails methylated at K9 are able to recruit the H3K9 methyltransferases GLP and ESET, as well as DNA methyltransferase 3A more efficiently, hereby contribute to gene repression.

### Note

During preparation of this manuscript, another group reported the MPP8-K9me3 complex [46]. Cheng and colleagues also observed that hMPP8 chromodomain formed homodimer both in solution and in crystal structures. The recognition mode between hMPP8 chromodomain and methylated histone H3K9 peptide in their complex structure is almost the same as that observed by us. We both found that hMPP8 cannot recognize methylated H3K27 and the binding affinity of hMPP8 chromodomain to H3K9me3 is similar.

## Materials and Methods

### Protein expression and purification

The chromodomain of human MPP8 (residue 56–116) was inserted into a pET28a-MHL vector via ligase-independent cloning. The recombinant protein was expressed in BL21 (DE3) Codon plus RIL (Stratagene). Cells were grown at 37°C to OD600 of approximately 6 and protein expression was induced by 0.1 mM IPTG for another 16 hours 15°C. Cells were collected by centrifugation and resuspended in lysis buffer (20 mM Tris-HCl, pH 8.0, 500 mM NaCl, 0.4% NP40, 0.5 mM TCEP, 5 mM immedazole, 20 ul Benzonase, and protease inhibitors). The resuspended cells were lysed by sonication and centrifugated at 16000 rpm for 60 minutes at 4°C. After centrifugation, the supernatant was passed through a Ni-NTA nickel-chelating column (Qiagen) equilibrated with lysis buffer and the column was extensively washed with washing buffer (20 mM Tris-HCl, pH 8.0, 500 mM NaCl, 25 mM immidazole, and 0.5 mM TCEP). Target protein was eluted with buffer (500 mM NaCl, 50 mM Tris, pH 8.0, 250 mM imidazole) for 3 column volumes. His-tag was removed by TEV protease. After digestion, protein sample was further purified by a HiLoad 16/60 Superdex 200 size exclusion column (GE healthcare).

### Protein Crystallization, X-ray diffraction data collection and structure determination

Before crystallization, the protein was concentrated to 26 mg/ml as stock in −80°C. Crystals of hMPP8 chromodomain were obtained by the hanging drop vapour diffusion method at 18°C in a buffer containing 25%PEG400, 0.2 M MgCl2, 0.1 M Hepes 7.5. For crystallization of complex, H3K9me3 peptide was mixed with hMPP8 chromodomain in an 8∶1 molecular ratio, then the mixture was crystallized using the hanging drop vapour diffusion method at 18°C. hMPP8-H3K9me3 complex was crystallized in a buffer containing 35% PEG2000-MME. Before flash-freezing crystals in liquid nitrogen, crystals were soaked in a cryoprotectant consisting of 100% reservoir solution and 15% glycerol.

Diffraction data were collected at beamline 19ID of the Advanced Photon Source (Argonne, Illinois). Data were reduced using the HKL suite [47]. Structures were solved by molecular replacement using the structure of human chromobox homolog 3 chromodomain (PDB ID: 3DM1) as template and refined with REFMAC [48]. The peptide ligands were automatically traced with BUCCANEER [49]. Interactive model rebuilding and validation were performed with COOT [50] and the MOLPROBITY server [51], respectively. The quality of the structure models was analyzed with the PROCHECK program [52]. The coordinates and structure factors have been deposited to the RCSB Protein Data Bank with accession numbers of 3LWE and 3R93. Details can be found in table 1.

### Surface Plasmon resonance (SPR) assay

The binding affinity of hMPP8 chromodomain and histone peptides were determined at 14°C using BIAcore3000 instruments. The biotinylated peptides were immobilized on a streptavidin-coated biosensor chip (SA-Chip). All experiments were carried out in the continuous-follow buffer (150 mM NaCl, 20 mM Tris, pH 8.0, 1 mM DTT). The injected protein sample was flowed for 3 min over the peptide coated SA-Chips at a follow rate of 30 ml/min and the change of response unit (RU) was recorded. Protein dissociation was monitored for 3 min by following the continuous-follow buffer at a follow rate of 30 ml/min over the SA-Chips. The KD was determined by global nonlinear regression fitting of the association and dissociation curves according to the Langmuir binding isotherm model.

## Supporting Information

Figure S1 **hMPP8 specifically recognizes methylated H3K9 rather than H3K27.** (A) A mutant structure model for hMPP8 binds to KAARK(me3)S histone motif. Gln5 and Thr6 of the QTARK^9^S motif were mutated to KA in this model. (B) Histone H3K27 peptide pulldowns with proteins of wild type hMpp8 chromodomain and indicated mutants, respectively. (C) Overall structure of the model for hMPP8 chromodomain in complex with histone motif KAARK(me3)S generated by the program HADDOCK. Motif KAARK(me3)S was comparable to methylated H3K9 peptide with an RMSD at 0.4 Å. (D) Superposition of the Trimethyllysine binding cage of the Docking model (green: chromodomian, cyan: KAARK(me3)S motif) with that of hMPP8 chromodomain in complex with methylated histone H3K9 peptide (yellow: chromodomain, pink: peptide). (E) Determination of the aggregation state of the hMpp8 chromodomain either in the presence or absence of histone H3K9me3 peptides. Molecular mass was measured by size exclusion. (F) Binding affinity of hMPP8 chromodomain to H3K4me3 (left panel, measured by ITC method) and H3K27me3 peptide (right panel, measured by SPR method).
(TIF)

Figure S2 **Two potential models of Mpp8 binding nucleosomes** ***in vivo*** **.** (A) hMPP8 homodimer binds to two H3K9me3 tails on the same nucleosome. (B) hMPP8 homodimer binds to two H3K9me3 tails on two spatially adjacent nucleosomes.
(TIF)
