# Supplementary figures and images for: Structural Basis for Specific Binding of Human MPP8 Chromodomain to Histone H3 Methylated at Lysine 9
Source: PLoS One. 2011 Oct 12;6(10):e25104. doi: 10.1371/journal.pone.0025104 (PMC3192050; doi:10.1371/journal.pone.0025104)

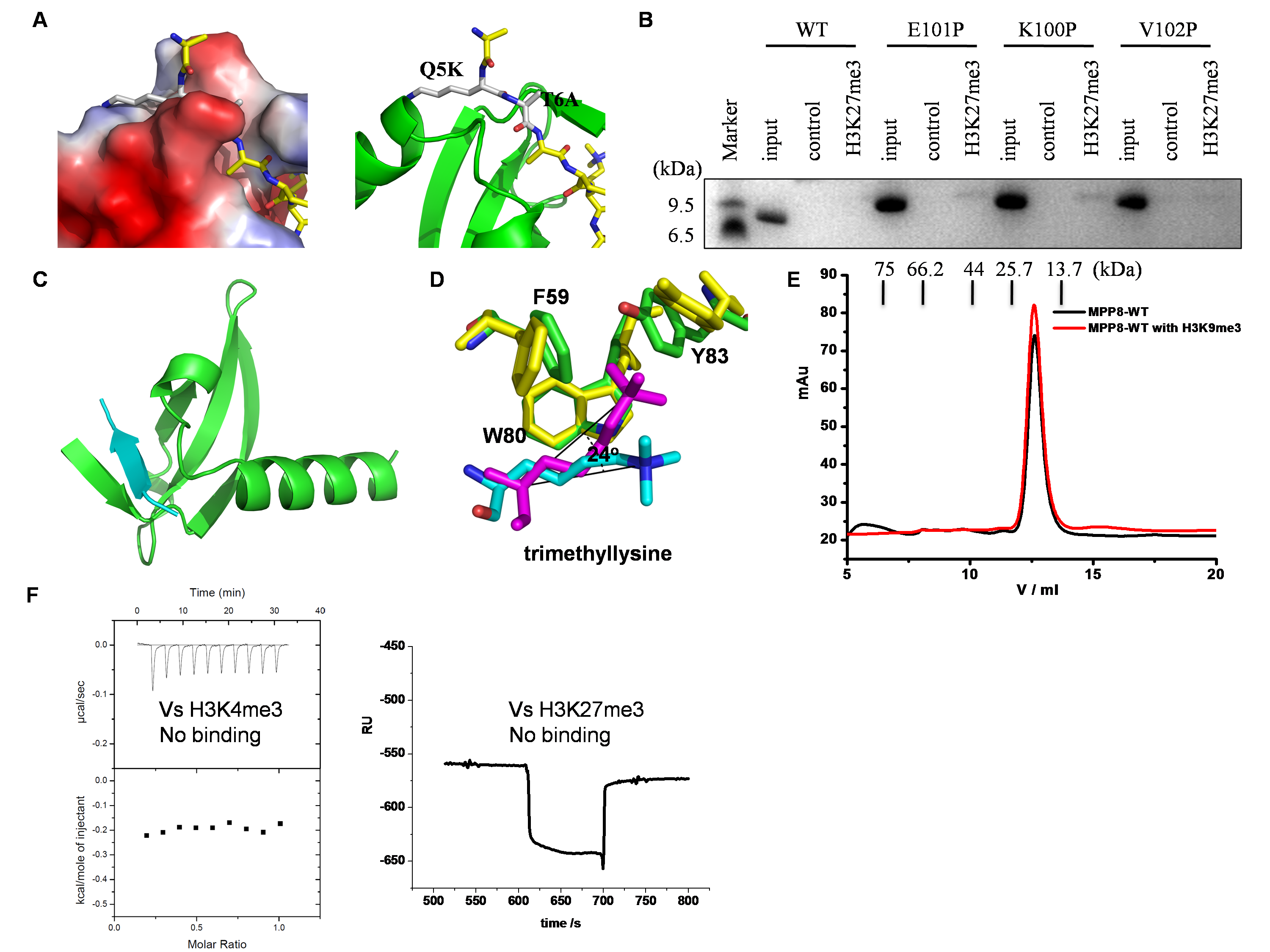

Supplement: Figure S1 — hMPP8 specifically recognizes methylated H3K9 rather than H3K27. (A) A mutant structure model for hMPP8 binds to KAARK(me3)S histone motif. Gln5 and Thr6 of the QTARK9S motif were mutated to KA in this model. (B) Histone H3K27 peptide pulldowns with proteins of wild type hMpp8 chromodomain and indicated mutants, respectively. (C) Overall structure of the model for hMPP8 chromodomain in complex with histone motif KAARK(me3)S generated by the program HADDOCK. Motif KAARK(me3)S was comparable to methylated H3K9 peptide with an RMSD at 0.4 Å. (D) Superposition of the Trimethyllysine binding cage of the Docking model (green: chromodomian, cyan: KAARK(me3)S motif) with that of hMPP8 chromodomain in complex with methylated histone H3K9 peptide (yellow: chromodomain, pink: peptide). (E) Determination of the aggregation state of the hMpp8 chromodomain either in the presence or absence of histone H3K9me3 peptides. Molecular mass was measured by size exclusion. (F) Binding affinity of hMPP8 chromodomain to H3K4me3 (left panel, measured by ITC method) and H3K27me3 peptide (right panel, measured by SPR method). (TIF) [file pone.0025104.s001.tif]

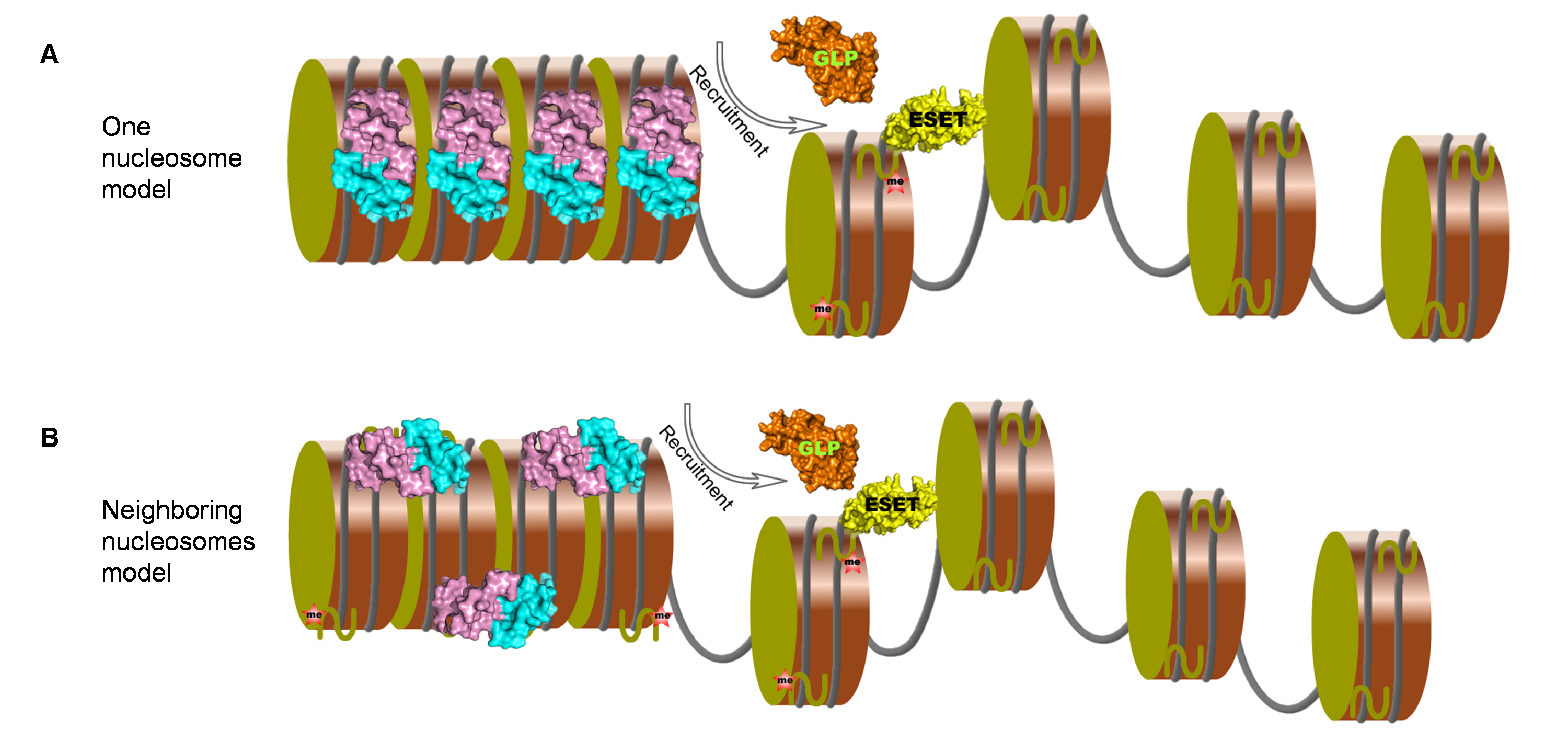

Supplement: Figure S2 — Two potential models of Mpp8 binding nucleosomes in vivo . (A) hMPP8 homodimer binds to two H3K9me3 tails on the same nucleosome. (B) hMPP8 homodimer binds to two H3K9me3 tails on two spatially adjacent nucleosomes. (TIF) [file pone.0025104.s002.tif]
